# Immediate and Late Effects of Early Weaning on Rat Gastric Cell Differentiation

**DOI:** 10.3390/ijms21010196

**Published:** 2019-12-27

**Authors:** Melissa Teles Silva, Kethleen Mesquita da Silva, Isadora Campos Rattes, Gizela Maria Agostini Zonta, Aline Vasques da Costa, Raquel Galvão Figueredo Costa, Ludimila Karen Cordeiro Nogueira, Daniela Ogias, Patricia Gama

**Affiliations:** Department of Cell and Developmental Biology, Institute of Biomedical Sciences, University of Sao Paulo, Sao Paulo 05508-000, Brazil; melissateles@yahoo.com.br (M.T.S.); kethms@usp.br (K.M.d.S.); isadora.rattes@usp.br (I.C.R.); gizela.zonta@usp.br (G.M.A.Z.); alinevasques@usp.br (A.V.d.C.); raquel.galfig@gmail.com (R.G.F.C.); cnogueira.ludimila@gmail.com (L.K.C.N.); daniela.ogias@gmail.com (D.O.)

**Keywords:** mucous neck cells, zymogenic cells, parietal cells, breastfeeding

## Abstract

*Background*: Gastric glands grow and cells reach differentiation at weaning in rats. By considering that early weaning (EW) can affect the timing of development, we aimed to compare molecular and cellular markers of differentiation in pups and adults. *Methods*: Wistar rats were separated into suckling-control (S) and EW groups at 15 days. Stomachs were collected at 15, 18, and 60 days for RNA and protein extraction, and morphology. *Results*: After EW, the expression of genes involved in differentiation (*Atp4b*, *Bhlha15* and *Pgc*) augmented (18 days), and *Atp4b* and *Gif* were high at 60 days. EW increased the number of zymogenic cells (ZC) in pups and adults and augmented mucous neck cells only at 18 days, whereas parietal and transition cells (TC) were unchanged. *Conclusions*: EW affected the gastric mucosa mostly in a transient manner as the changes in gene expression and distribution of differentiated cells that were detected in pups were not fully maintained in adults, except for the size of ZC population. We concluded that though most of EW effects were immediate, such nutritional change in the infancy might affect part of gastric digestive functions in a permanent manner, as some markers were kept unbalanced in the adulthood.

## 1. Introduction

In adult animals, the proliferation, differentiation, migration and death of gastric epithelial cells are balanced to allow mucosa renewal and the maintenance of gland architecture and functions [1,2,3,4,5] (for a review of the cellular plasticity nomenclature see [6]). Any disturbance of these steps disrupts the equilibrium and can lead pathologies and diseases [7,8,9,10]. During postnatal development, the kinetics processes listed above are driven towards growth, and they require distinct regulatory mechanisms [11,12,13,14]. Interestingly, some pathological conditions recapitulate developmental features, and so, it is essential to understand the gastric postnatal ontogenesis and the origin of cell lineages, and to explore the effects of disturbances during growth and their late consequences in adulthood [15,16,17,18].

During postnatal development, gastric epithelial cells proliferate along the gland [1,11,13] without a clear distinction of the stem cell compartment, in contrast to the organization in the adult that shows an active proliferative area at isthmus-neck interface [1,15,19,20,21,22,23] and a slow cycling reservoir at the base [5,9,16,24]. The first cell lineages to expand in number and activity are surface mucous and parietal cells (PC), which are followed by mucous neck (MNC), zymogenic (ZC) and endocrine cells [1,2,25,26,27]. PC are identified as round eosinophilic cells that use a membrane canaliculi system to pump H^+^ and transport Cl^-^, but they also synthesize the transforming growth factors alpha and beta (TGF-*α* and TGF-*β*) [28,29]. Moreover, PC are important to the organization of the active stem cell niche and the whole architecture of the gastric gland [17,23,30], and thus, they physically influence the up- and downwards movements of the other cell populations [23]. While migrating from isthmus to neck region, MNC differentiate, and then part of them expresses a mixture of markers and shows an intermediary phenotype (transition cells, TC) that will be finally differentiated into ZC [4]. MNC are characterized by the transcription of *Muc6* and spasmolytic factor (also described as trefoil factor 2—TFF2) [31], and during the transition to ZC, *Bhlha15* gene codifies transcription factor Mist1 to organize mucous-serous changes [4,32,33] that prepare ZC to synthesize and secrete pepsinogens (*Pga5* for pepsinogen A until weaning, and *Pgc* for pepsinogen C after weaning and throughout aging) [34,35] and the gastric intrinsic factor (GIF) in rodents [23,36].

As mentioned above, during postnatal development, the imbalance between cell proliferation and death results in growth, and disturbances during this period, especially those affecting breastfeeding can change these processes. Previously, we interfered in gastric growth regulation during suckling through the induction of fasting [11], neonatal-maternal separation [37] and early weaning [12,14,28,29,38,39,40,41]. Specifically for early weaning, we showed that MNC and ZC are sensitive to the abrupt substitution of milk by chow [28,38], and we also demonstrated that it immediately increases gastric epithelial cell proliferation through EGFR signaling pathways [28,39,40], ghrelin [41] and corticosterone activity [13,38]. Finally, although some of the early weaning-regulated responses in MNC and ZC persist throughout the first postnatal month, we still do not know whether and how they are maintained in adulthood.

Currently, our specific hypothesis considered that if early weaning interfered in cell proliferation and accelerated the maturation of the gastric gland during the first postnatal month in rats, then it could affect directly the regulatory mechanisms that coordinate the differentiation of cells and their functions both during ontogenesis and in adulthood. Such changes in developing period and adult life might clinically affect gastric functions. Therefore, because early weaning can alter essential processes involved in the coordination of gastric growth, we currently aimed to compare its effects on molecular and cellular markers of differentiation in rat pups and adults in order to characterize immediate and late responses of cell populations that are essential for gastric glands. Our results showed that whereas early weaning rapidly affected gene expression and distribution of gastric cells in pups, only some of these responses were maintained in adults. Therefore, we suggested that in the gastric mucosa most of early weaning-induced changes was transient, but part of them was maintained and might influence gastric cells in a permanent manner in adulthood.

## 2. Results

### 2.1. Early Weaning and Body Mass

**Figure 1:**
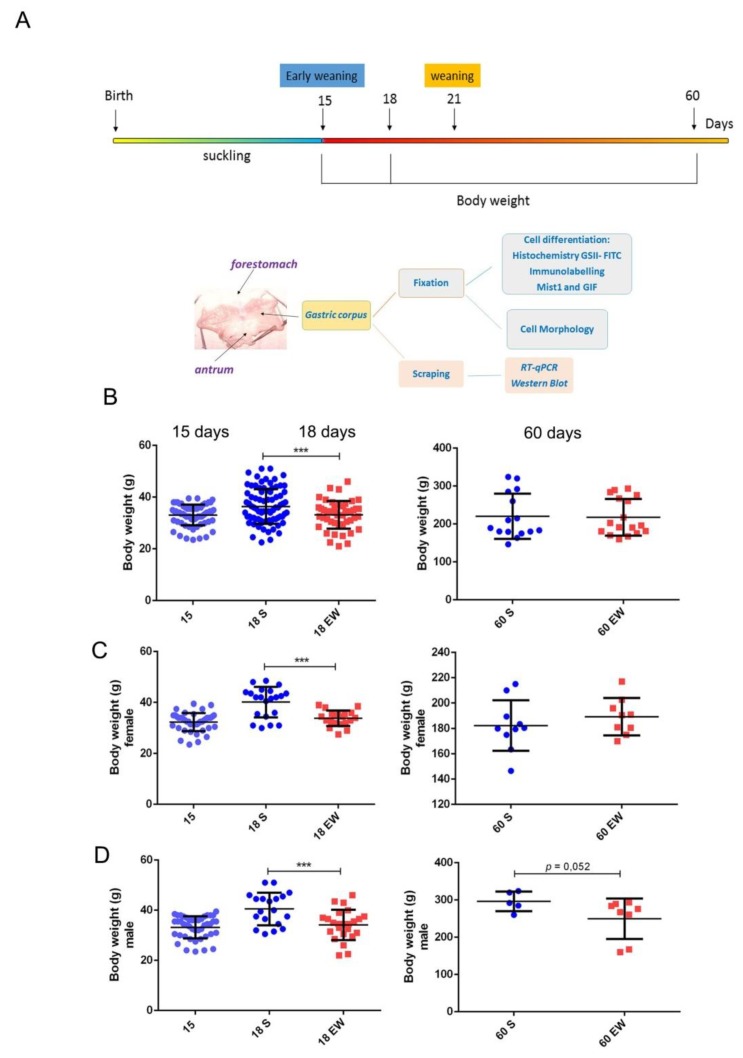
Immediate and late effects of early weaning on body weight gain. (**A**) Experimental design for gastric samples collection and body weight control for comparison of suckling (S) and early weaning (EW) groups at 15, 18, and 60 days. (**B**) Body weight (g) was reduced immediately after early weaning (18 days), and differences decreased (60 days) when all rats were studied. (**C**,**D**) Data was analyzed for females and males, separately. The body weight of each rat in S (blue) or EW (red) condition is represented individually. Means ± SD is also indicated for each group. Results were compared after unpaired Student *t* test between S and EW at one age as *** *p* < 0.0001.

Changes in quality and origin of nutrients during suckling-weaning period affect directly the epithelial cells that cover the gastrointestinal tract [12,28,29,37,38,39], and to further investigate the responses in the gastric mucosa, we induced EW at 15 days (Figure 1A). At first, by considering that EW influences body weight gain in rats [38,41], we studied its effects in pups and adults (Figure 1B). We found that immediately after the onset of EW (18 days), animals showed a 17.6 % reduction in body weight when compared to S-pups (*p* < 0.05), but as they grew, such difference progressively decreased and S and EW groups were similar at 60 days (Figure 1B). As analyses were performed with males and females, and throughout aging, male rats gained more mass than females, we also studied them separately. Though we observed a similar recovery response in body weight gain, we detected that EW females, that had been also smaller as pups, recovered body mass and gained weight at 60 days (3.8 % higher for EW vs. S group; *p* = 0.2) (Figure 1C). Conversely, EW males maintained a lower mass until adulthood (reduction of 15.6% at 60 days; *p* = 0.052) when compared to animals from S group (Figure 1D).

### 2.2. Ontogenic Expression of Genes That Regulate the Differentiation of Gastric Epithelial Cells

**Figure 2:**
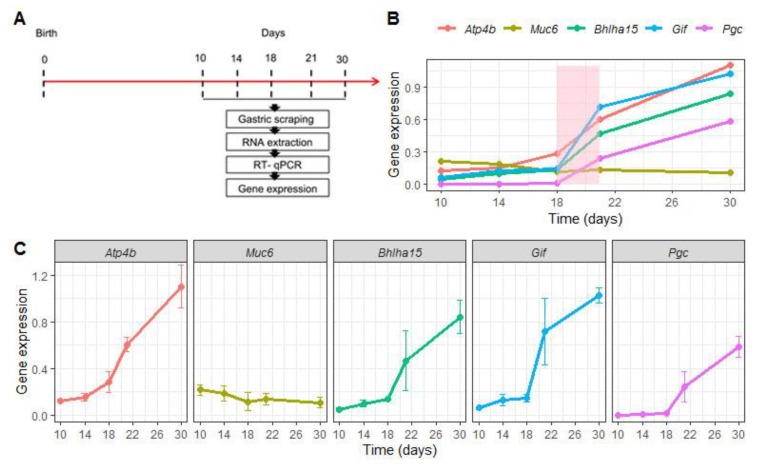
Expression of regulatory marker genes changes during gastric growth. (**A**) Experimental design for RNA isolation at 10, 14, 18, 21 and 30 days in suckling rats. (**B**) Comparative expression of *Atp4b*, *Muc6*, *Bhlha15*, *Gif* and *Pgc*, which are respectively important for differentiation and function of parietal cells (*Atp4b*), mucous neck cells (*Muc6*), and zymogenic cells (*Bhlha15*, *Gif* and *Pgc*). Levels were represented as fold induction of internal control (*Gapdh*) throughout the first postnatal month. The weaning period is colored (light pink) to identify the natural and ontogenic increase in expression of the genes studied. Each dot represents the means ± SD at each age. *n* = 4–5 animals. Technical replicates: *n*= 2. (**C**) Expression levels of each gene is separately demonstrated. One- way ANOVA was used to compare the variation throughout the first postnatal month; *p* < 0.05 was detected for all genes, except for *Muc6* analyses that did not indicate changes.

Before evaluating the effects of EW on the genes that regulate differentiation and function of gastric epithelial cells, we characterized their expression during the first postnatal month. To that end, samples were collected at 10, 14, 18, 21 and 30 days from S animals (Figure 2A). We compared *Atp4b*, *Bhlha15*, *Pgc* and *Gif* mRNA levels throughout this period and we observed that they progressively augmented (Figure 2B,C), but more importantly, we found that weaning represented an important stage during the ontogenic increase (Figure 2B). When these marker genes were analyzed separately, we noted that their increase was significant when the ages were compared (*p* < 0.05) (Figure 2C), except for *Muc6* expression that was not altered (Figure 2B,C). Such response might indicate a constant genetic program, though MNC morphologically differentiate during the third postnatal week [2,25,28].

### 2.3. Early Weaning Changes Molecular Markers of Differentiation and the Distribution of Gastric Cells

**Figure 3:**
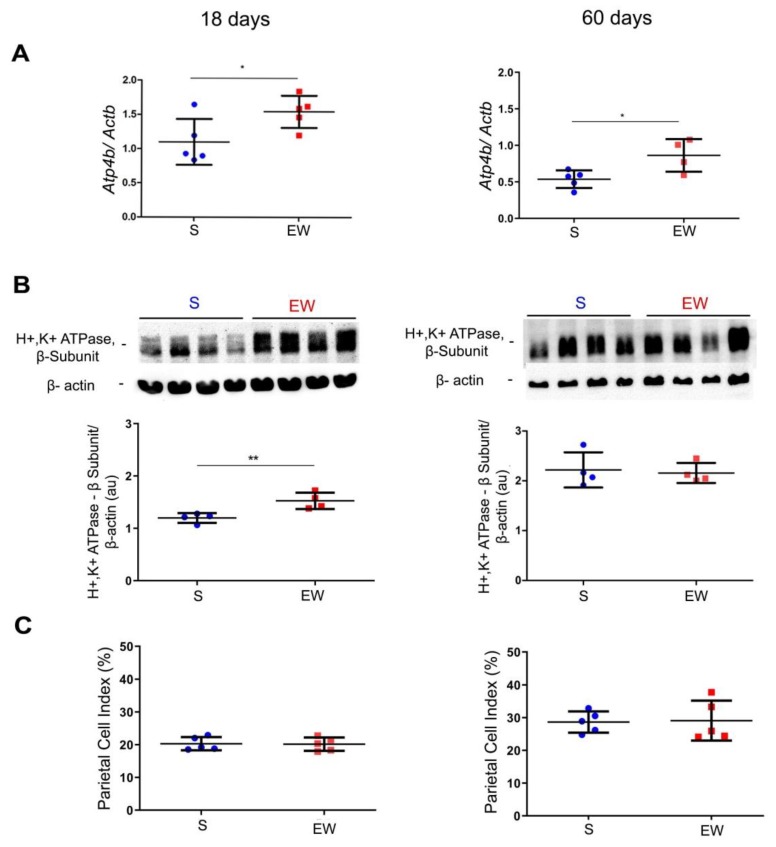
Early weaning effects on markers of parietal cell (PC) activity and distribution. The gastric mucosa was studied in suckling (S) and early-weanling (EW) groups at 18 and 60 days. (**A**) Expression of *Atp4b* after RT-qPCR represented as fold induction of internal control (*Actb*). *n* = 3–5 per group. Technical replicates: *n* = 2. (**B**) Protein levels of H+/K+-ATPase proton pump (52 kDa) were determined by integrate optical densitometry (arbitrary units) in immunoblots and compared to the internal control protein (*β*-actin, 42 kDa). *n* = 4 per group. Technical replicates: *n* = 2. (**C**) The PC index (%) was obtained by counting the number of parietal cells among 1000 epithelial cells under light microscopy in HE stained sections. *n* = 5 per group. Individual results for S (blue) and EW (red) rats are represented and means ± SD is indicated in each group and age. After unpaired Student *t* test between groups in each age: * *p* < 0.05 and ** *p* < 0.01.

Once the ontogenic profile was identified in S pups, we studied the rat gastric mucosa at 18 and 60 days to detect immediate and late effects of EW on genes involved in organization and differentiation of parietal, mucous neck and zymogenic cells and the distribution of these populations in the gastric mucosa. Parietal cells differentiate early in development [30] and they are important to the organization, structure and function of gastric glands [18,42,43]. We investigated the levels of *Atp4b* and H^+^K^+^/ATPase pump, and the distribution of PC (as PC index) in pups and adult rats submitted to EW. We observed that *Atp4b* expression augmented after EW both at 18 and 60 days (*p* < 0.05) (Figure 3A). The protein levels of H^+^K^+^/ATPase pump increased at 18 days (*p* < 0.05) (Figure 3B), but such response was not maintained at 60 days. In histological sections stained with hematoxylin and eosin, parietal cells are easily observed by their round eosinophilic morphology and after their quantification, we did not find differences in PC indices between S and EW groups at 18 and 60 days (Figure 3C).

**Figure 4:**
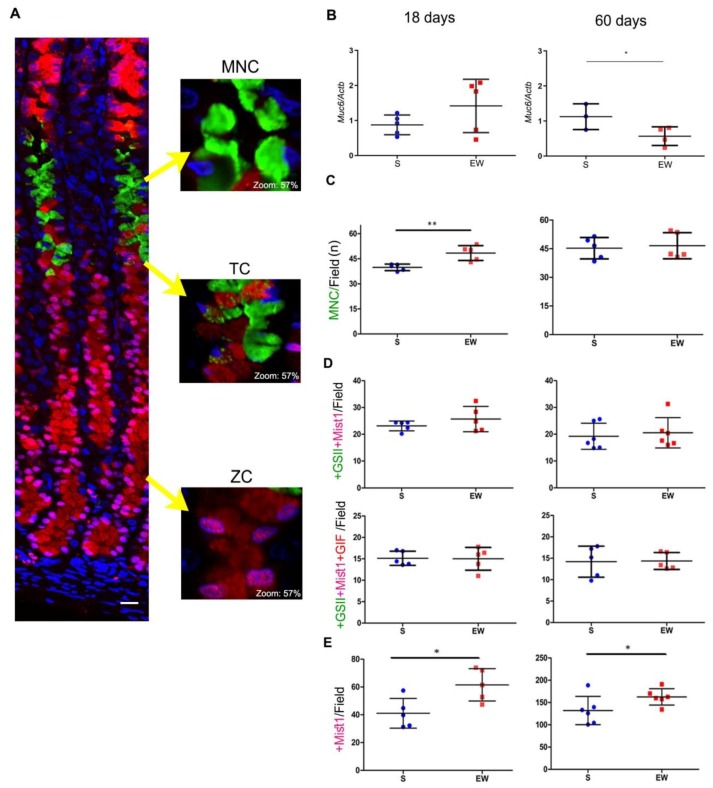
Early weaning affects gastric cell distribution through transient and long- lasting effects on MNC, TC and ZC in the gastric mucosa of S and EW rats at 18 and 60 days. (**A**) Representative photomicrographs of the gastric mucosa illustrate the distribution of mucous neck cells (MNC: +GSII/FITC), transition cell (TC: +GSII/FITC+Mist1/Cy3 or +GSII/FITC +Mist1/Cy3+GIF/AlexaFluor 633) and zymogenic cells (ZC: +Mist1/Cy3). Nuclei were counterstained with DAPI. Representative images were acquired using confocal microscopy (Zeiss LSM 780-NLO). Scale bar = 50 μm. The insets show in detail the morphology and labeling of each cell type; digital zoom was set at 57%. (**B**) Expression of *Muc6* after RT- qPCR represented as fold induction of internal control (*Actb*). *n* = 3–5 per group. Technical replicates: *n*= 2. (**C**–**E**) The quantification of each cell type/field/animal was performed for (**C**) as MNC/field/animal; (**D**) TC/field/animal after counting double- labeled (+GSII/FITC+Mist1/Cy3) or triple- labeled (+GSII/FITC+Mist1/Cy3+GIF/AlexaFluor 633) and (**E**) ZC/field/animal. To determine the density of each cell type, five fields/rat were used, considering the central area in corpus region and longitudinally sections glands. For quantification, images were acquired under fluorescence microscopy (Axioscope 2, Zeiss). Individual results are represented for S (blue) and EW (red) rats and means ± SD is indicated in each group and age. Unpaired Student *t* test was used and * *p* < 0.05, ** *p* < 0.01.

Next, we compared the effects of early weaning on mucous neck, transitory and zymogenic cells in 18- and 60-day-old rats (Figure 4). First, we registered a non-significant increase of *Muc6* expression at 18 days (Figure 4B) and the inversion of response (decrease) at 60 days (Figure 4B) (*p* < 0.05). After multi-labeling under fluorescence microscopy, we observed the MNC in the gland neck as triangular cells containing glycoprotein granules that reacted with GSII lectin- FITC (Figure 4A). The number of MNC/field increased after EW at 18 days (Figure 4C) (*p* < 0.01), and this density did not change at 60 days (Figure 4C).

As a part of MNC population originates ZC, a transitory phenotype can be visualized (TC) in a narrow compartment between the neck and base of the gland (Figure 4A). In order to study TC, we used multi-labeling reactions that characterized the co-localization of mucin granules (+GSII) with transcriptional factor Mist1 (+Mist1) and gastric intrinsic factor (+GIF) (Figure 4A). As an important step in the transition from MNC to ZC involves the transcription of Mist1 in +GSII- cells, we determined the distribution of double-labeled cells/field, and observed similar numbers of +GSII+Mist1 cells/field in the mucosa at 18 and 60 days (around 20 cells/field), regardless dietary pattern (S or EW) (Figure 4D). To identify whether this population was starting to synthesize ZC products, we determined the distribution of triple-labeled cells/field and verified similar numbers of +GSII+Mist1+GIF/field (around 15 cells/field) in the mucosa of S and EW rats at both ages (Figure 4D). Zymogenic cells are small and confined to the base of the gland and we registered those that were immunolabeled for Mist1 (Figure 4E), as it is the transcriptional factor necessary for the whole population, independent of their secretory activity. After counting the number of ZC/ field, we demonstrated that EW increased ZC at 18 and 60 days (*p* < 0.05) (Figure 4E), indicating the maintenance of effects on the distribution of this population in adults rats. 

**Figure 5:**
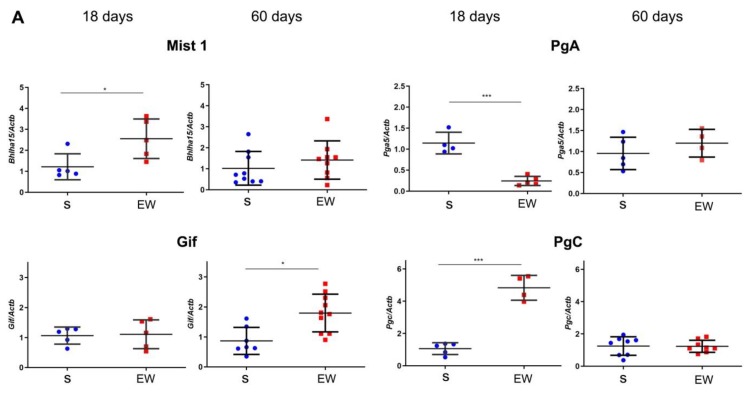

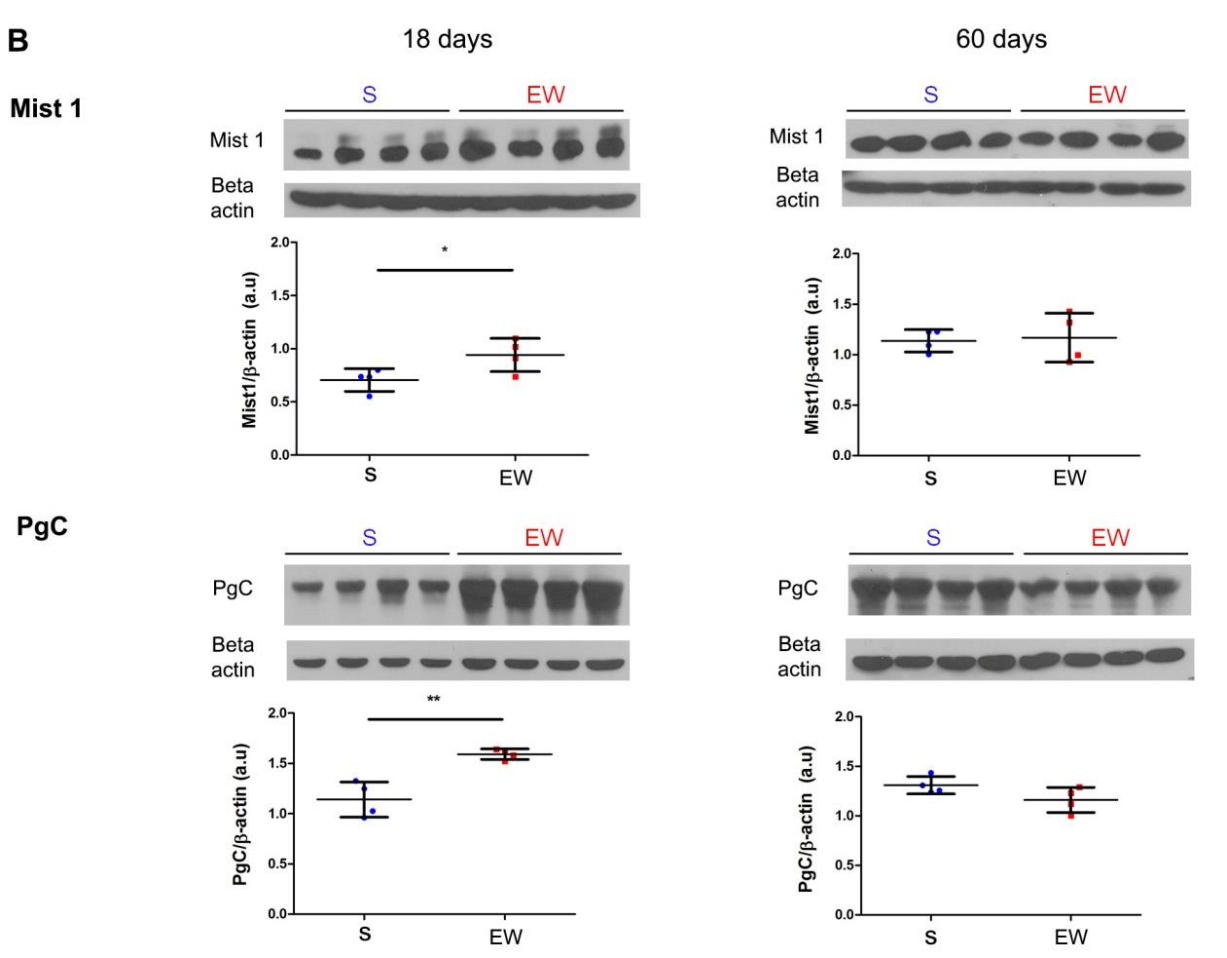
Early weaning alters the expression of regulatory genes and protein levels in zymogenic cells in the rat gastric mucosa. (**A**) Expression of *Bhlha15*, *Gif*, *Pga5*, *Pgc* was compared in gastric mucosa of S and EW rats at 18 and 60 days and levels are indicated as fold induction of internal control (*Actb*). Biological replicates: *n* = 3–10 animals. Technical replicates: *n* = 2–3. (**B**) tissue lysates from gastric mucosa were used to detect the transcription factor Mist1 (22 kDa) and pepsinogen C (42 kDa) at 18, and 60 days. Protein levels were determined by integrate optical densitometry (arbitrary units) after immunoblots and compared to the internal control protein (*β*-actin, 42 kDa). Individual results are represented for S (blue) and EW rats (red) and means ± SD is indicated in each group and age. After unpaired Student *t* test between groups/age: *** *p* < 0.001; * *p* < 0.05, ** *p* < 0.01.

Next, we evaluated the expression of markers that regulate the differentiation and function of zymogenic cells in the gastric gland, and we found that EW immediately augmented the expression of *Bhlha15* (*p* < 0.05), and *Pgc* (*p* < 0.001), whereas *Gif* levels were similar to S group (Figure 5A). Interestingly, *Pga5* mRNA concentration, which naturally decreases with aging, was drastically reduced after EW in pups (*p* < 0.001) (Figure 5A). As some genes might be primed by dietary changes during development [37,38], we compared the responses at 60 days. We found that expression was similar between S and EW groups for most of genes (Figure 5A), except for *Gif* that maintained higher levels in EW group when compared to S rats (Figure 5A) (*p* < 0.05). 

Because *Bhlha15* and *Pgc* genes that encode Mist1 and pepsinogen C were affected by EW in pups, we checked their protein content after immunoblots. We noticed that after EW, Mist1 and pepsinogen C protein levels increased at 18 days (Figure 5B) (*p* < 0.05), whereas at 60 days, their contents were not altered significantly (Figure 5B).

**Figure 6:**
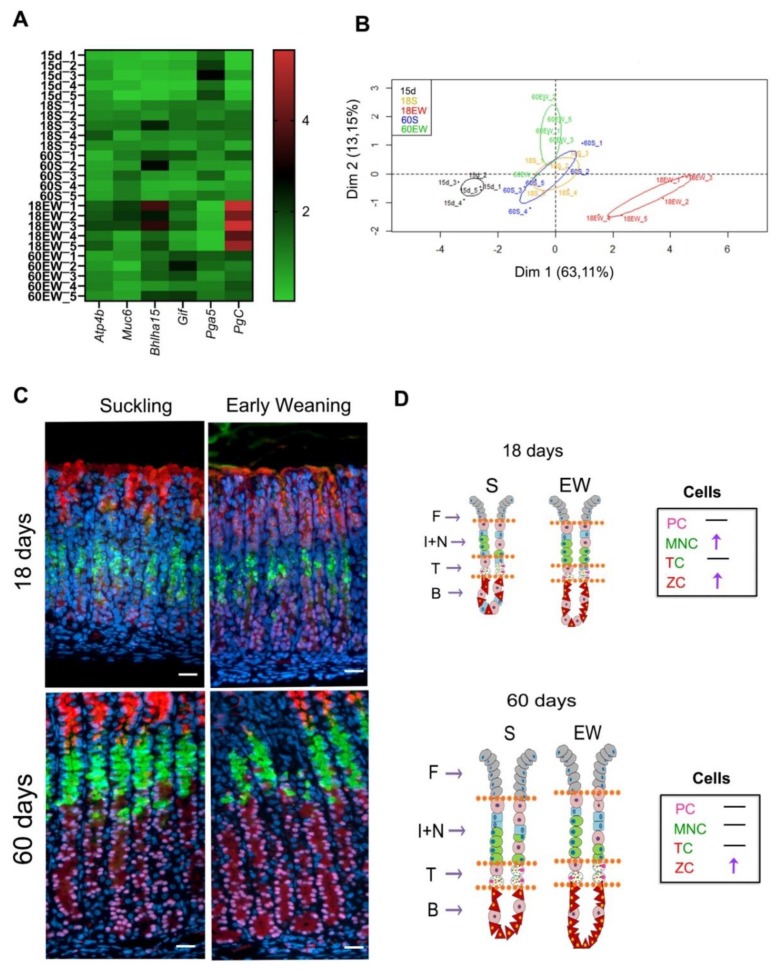
Summary of early weaning effects on the differentiation of gastric cells: transient and permanent responses. (**A**) Heat map of gastric mucosa gene expression data at different ages. Red represents increased gene expression, while green represents reduced gene expression. (**B**) Principal Component Analysis (PCA) of gastric mucosa gene expression data. PCA two-dimensional scatter plot represent the differential gene expression patterns of suckling and early-weaning rats at different ages. Axis: X = Dim1-Principal Component 1 (63.11% variance); Y = Dim2-Principal Component 2 (13.15% variance). (**C**) Representative images from the gastric mucosa of suckling and early- weanling rats at 18, and 60 days. Sections were used for multiple labeling to detect: mucous granules (GSII/FITC) in MNC and TC; Mist1/Cy3 in the nuclei of TC and ZC; GIF/AlexaFluor 633 in serous granules in TC and ZC. Nuclei were counterstained with DAPI. Images were obtained under fluorescence microscope, using separated filters, and they were combined through Zen Software. Acquisition at X 40. Bar = 50 μm. (**D**) Schematic distribution of PC, MNC, TC and ZC in the gastric mucosa (F—foveola; I + N—isthmus and neck; T—transition; B—base).

Finally, we summarized the data obtained for gene expression and distribution of cells and compared the effects of EW on the differentiation of gastric mucosa (Figure 6). Results obtained for 15-day-old pups were added to a heat map and the principal component analyses (PCA) in order to evaluate the profile of gastric molecular markers according to growth and dietary change. From the panel genes studied, we detected that *Pga5* was high at 15 days, as expected, whereas *Bhlha15* and *PgC* increased with aging (Figure 6A). Interestingly, early weaning augmented the expression of *Atp4b*, *Muc6*, *Bhlha15*, *Gif* and *PgC,* and the highest scores were registered at 18 days (Figure 6A). In addition, as we aimed to identify how EW affected the gastric mucosa and whether the effects were maintained until adulthood, we used PCA analysis to correlate the results on gene expression. We found distinct clusters of data that were separated along the first principal component (Dim 1), as those from 15-day-old pups were separated from animals in S groups at 18 and 60 days, which were similar among themselves (Figure 6B). EW triggered an immediate and strong response in 18-day-old rats, which were identified as a cluster to the right along Dim1 (Figure 6B). In the adult gastric mucosa, the expression of molecular markers could still be distinguished from S groups, but in a different magnitude (Figure 6B). 

When cellular distribution was compared between S and EW at both ages (Figure 6C,D), we noted that at 18 days, both MNC and ZC populations were altered, and at 60 days, only the number of ZC increased in EW group (Figure 6D). The transitory compartment was unaltered after EW at 18 and 60 days (Figure 6C,D).

## 3. Discussion

Our current study was designed to evaluate the gastric gland organization during rat postnatal development and to compare the immediate and long- term effects of early weaning on epithelial architecture. More specifically, we aimed to analyze whether the abrupt change of diet would alter the expression of regulatory markers for differentiation, morphology and distribution of mucous neck, transition, zymogenic and parietal cells, and we also investigated whether these elements would be primed to maintain the changes until adulthood.

Early weaning is a model that is used to mimic a social conduct, in which breastfeeding is neglected and ends up influencing parameters related to growth, physiology and behavior [44], and in that sense, body mass gain is one of the first aspects to be analyzed. In the current study, we found an immediate reduction of weight at 18 days and recovery at 60 days, which confirmed previous studies that discussed these effects using the same [28,41,45] or similar EW protocols [46,47,48]. However, we detected a variation between males and females at 60 days, which is naturally registered, but after EW, whereas females fully recovered, males maintained lower weight when compared to S animals. Other reports demonstrated that ghrelin and leptin change after early weaning in females [49], and together with other hormones, they might be involved in the control of adiposity and food intake when diet is abruptly altered during infancy [50,51,52,53,54].

In attempt to contribute to the characterization of cell lineages during gastric postnatal development, we evaluated the expression of hallmark genes that are important for terminal differentiation and activity in parietal (*Atp4b*), mucous neck (*Muc6*) and zymogenic cells (*Bhlha15*, *Pgc* and *Gif*). Excepting for *Muc6*, which was continuously synthesized and might be under post-transcriptional control to regulate the content of mucin 6 during ontogenesis [38], the expression of these markers augmented during the first month, with an important increase by weaning period that might be determinant for the differentiation of cell lineages [4,25,34,55,56]. As suckling- weaning transition represents such an essential stage for gastric maturation [1,2,25,28,34,38], disturbances of feeding pattern are able to reprogram some steps and accelerate differentiation [28,38]. However, questions remained on whether and how long the changes would last. When parietal cells were studied, we observed that EW induced a high expression of *Atp4b* in pups, that was maintained in adults, whereas the protein levels of H^+^K^+^/ATPase only increased at 18 days and the distribution of cells was unaltered by dietary change. A previous report showed that EW triggers different responses in size and density of PC, depending on rat age [45], but our results agree when cell number is compared in adult animals. The roles of parietal cell to gastric gland organization have been debated and its importance in the control of migration of the other cell populations is evident [23]. We suggest that EW induced the expression of *Atp4b* both in pups and adults, priming this gene with a change, which did not reflect directly in function or cell number, possibly because post- transcriptional mechanisms might be down- regulating the proton pump and its activity.

By evaluating the effects of EW on secretory cells, we observed that in MNC, the expression of *Muc6* was unresponsive in pups and decreased in adults, suggesting a late regulation of transcription that might affect the cell activity in adulthood. Cell distribution was influenced by EW as it increased MNC number at 18 days, but at 60 days a there was similar density between S and EW groups. However, as we focused on the transition of MNC to ZC, that can occur without necessarily going through a proliferative step [8], we found that EW increased ZC distribution (Mist1 + cells) at 18 and 60 days. Previously, we had demonstrated that the disturbance of dietary pattern during postnatal development induces the amplification of ZC population at 30 days and the synthesis of pepsinogen C, and moreover, corticosterone contributes to these changes [37,38]. Also, EW disrupts TGFα- EGFR pathway by increasing signaling [28,39,40], which in transgenic animals [57] and Mènètrier’s disease [58] reduces ZC and PC number, respectively. Adding to these mechanistic cascades, the effects of EW on the size of ZC population might be attributed to changes in kinetics, especially increased proliferation in the gland, transition from MNC, and migration. So, our previous results showed that EW augmented *Muc6* transcription at 17 days and the number of MNC at 18 days, characterizing a transient response in activity and number of cells [38] derived from high proliferative rates described under this condition [12,39,40]. In that way, in pups, the immediate mechanism of EW would be to increase cell proliferation, which would expand MNC population to originate more ZC, without affecting PC. But as in adult rats, MNC were unaltered and ZC increased, we pored over transition cells and dissected the compartment between the neck and base of the gland.

Our results demonstrated that the size of transition compartment was not altered by EW, as the numbers of +GSII + Mist1/ and +GSII + Mist1 + GIF/field/animal did not change after dietary change. Interestingly, we also observed that the population in transition was the same through aging, which means that proportionally, in pups and adults, the transition compartment occupies and represents the same area in the gland. So, this is the first study to show that during postnatal development and in the adult mucosa, the differentiation of zymogenic cells was maintained, even after adverse conditions, such as the subtle change of feeding pattern. Moreover, we consider that as the production and secretion of pepsinogen C to the acid environment characterize the gastric function, they might be protected under any condition to guarantee digestion, i.e., the premier function in the organ. However, the EW-induction of ZC might be still derived from a higher influx of cells towards the base and the transition compartment would function as a gate, controlling such movement. Alternatively, as it was recently suggested for adult mice [59], changes in ZC population come mostly from self- renewal, independent of MNC-ZC model, and EW could have triggered this mechanism. Therefore, independent of the mechanism, ZC population is affected by early weaning, and such change can lead to modifications either of their regular physiological functions or of pathological conditions, as SPEM induction. Other studies are still necessary to identify how ZC population expands after EW and whether the effects trigger or facilitate diseases in adulthood.

The differentiation and functions of ZC depend directly on the expression of regulatory genes, and we observed the immediate and rapid response at 18 days, which is necessary to modify the secretory apparatus during MNC-ZC transition [4]. Because the process starts in the immature mucosa, transition cells might demand more *Bhlha15* and its encoded protein Mist1 soon after the nutrient change promoted by EW. In addition, *Pga5*, which identifies the immature pepsinogen at suckling period, was decreased by EW at 18 days, confirming that pepsinogen A was substituted by pepsinogen C, which is the polypeptide to be activated to pepsin. By comparing the responses to EW at 18 and 60 days, we found that in adults, both gene and protein levels were reversed and were like those detected in S rats. From the panel of ZC markers, *Gif* behaved similarly to *Muc6*, as it was not altered at 18 days and then increased 60 days, indicating that EW lately regulated the transcription of this gene. These results suggested that for most of genes involved in the maturation and function of ZC, EW induced a transient effect, but *Gif* was primed and might interfere in cell activity in adulthood.

In summary, we demonstrated that suckling- weaning is an important period in the regulation of the expression of marker genes in gastric epithelial cells and the distribution of mucous neck and zymogenic cells. We showed that early weaning rapidly affected gene expression and distribution of gastric cells in pups, but only some of these responses were maintained in adults. Therefore, by considering our specific initial hypothesis, we suggested that in the gastric mucosa most of early weaning-induced changes was transient, but part of them was maintained and might influence gastric cells in a permanent manner in adulthood. The resultant of these conditions might have different roles in developing animals and in adults, since they are determinant for the physiological growth and maturation, but they can also change the microenvironment for the induction of gastric diseases.

## 4. Materials and Methods 

### 4.1. Animals and Early Weaning

Specific-pathogen-free Wistar male and female rats were obtained from the Animal Facility at Institute of Biomedical Sciences (University of São Paulo) and transferred to the Animal Colony at Department of Cell and Developmental Biology (ICB-USP). Animals were maintained at 22 °C under 12-hour light and 12-hour dark cycles (lights on at 6.00 a.m.). Rats were mated, and pregnant females were housed in isolated cages on the 18th gestational day. Delivery was set as day 0 and litters were culled to 8–9 pups on the 3rd day. At 15 days, male and female pups were randomly assigned to suckling (S) or early-weanling groups (EW). Rats kept as S remained with the dam and regular weaning was performed at 21 days, whereas EW animals were separated from their mothers and placed in a small cage with hydrated powered chow and water *ad libitum* (Nuvilab CR-1, Quimtia S/A, Colombo, PR, Brazil). In order to mimic maternal care and guarantee chow consumption, twice a day, EW rats received food and water through disposable Pasteur pipettes, and were massaged to facilitate excretion of urine and feces. S pups were also manipulated daily. In order to avoid obesity and stressful conditions through isolation, litters were maintained with 6–9 pups/ dam or cage. Body weight was monitored and compared between S and EW groups, and considering the effects of EW on females and males, separately. Animals were euthanized at 15, 18, and 60 days, after anesthesia with ketamine and xylazine hydrochlorides at 1:1 (*υ*/*υ*) (0.5 mL/100g body weight) (Dopalen and Anasedan, Vetbrands^®^, Jacareí, SP, Brazil) at 10.00 a.m. The stomachs were excised and opened along the lesser curvature for sampling from corpus region (Figure 1A). For RNA and protein isolation, the mucosa was scraped and added respectively to 100 µL of RNALater^®^ (Life Technologies, Carlsbad, CA, USA) or 10 mM phenylmethylsulfonyl fluoride (PMSF) (Merck, Darmstadt, Germany) in 20 mM Tris-buffered saline (TBS). For morphological studies, the stomach was stretched on a corked and fixed in 4% or 10 % formaldehyde, as indicated below for light microscopy analyses.

### 4.2. RNA Isolation, cDNA Synthesis and RT-qPCR 

After isolating RNA with TRIzol (Invitrogen, Carlsbad, CA) combined to PureLink^®^ RNA Mini Kit (Invitrogen), we followed the protocol described before for cDNAs synthesis and quantitative PCR [38] with the primer sequences (Table 1) for SYBR Green technique (ontogenic analyses) and TaqMan^®^ probe assays (Table 2) (Thermo Fisher Scientific, Waltham, MA, USA) (S vs. EW comparisons), as indicated. Data was collected and results were calculated using 2^−∆∆Ct^ method [60].

### 4.3. Protein Extraction and Western Blot

Proteins were extracted after tissue lysis with RIPA buffer. After quantification through Bradford method [37,61], thirty µg of proteins were separated into 12% SDS-PAGE for blotting onto nitrocellulose membranes (GE Healthcare, Buckinghamshire, UK), as described before [62]. Membranes were blocked in TBS superblock (AG41422, Pierce, Rockford, IL, USA) (10 min at room temperature) (RT), and the following primary antibodies (Santa Cruz Biotechnology, Santa Cruz, CA, USA) were used through SNAP incubation apparatus (SNAP i.d.™, Millipore, Bedford, MA): goat anti-pepsinogen C (0.1 µg/mL) (I-19; sc-51188) and mouse monoclonal anti-Mist1 (0.2 μg/mL) (6E8; sc-80894). Membranes for detection of H^+^/K^+^-ATPase proton pump were blocked in 5% non-fat milk -TBS and incubated overnight 4 °C with monoclonal rabbit anti-H^+^/K^+^-ATPase *β* subunit (119103, Calbiochem, San Diego, CA, USA). Monoclonal anti-*β*-actin (1 µg/mL) (A5541; Sigma Aldrich, St. Louis, MO) was used as internal loading control. Secondary rabbit anti-goat (0.53 µg/mL), goat anti-mouse (0.26 µg/mL) goat anti-rabbit (0.26 µg/mL) (305035003, 115035003 and 111035003, Jackson Immuno Research Laboratories, Inc., West Grove, PA, USA) conjugated to peroxidase were used to specifically detect the antibodies above (10 min at RT or 1 h at RT). Reactions were developed with ECL Prime (Mist1) or ECL (pepsinogen C, H^+^/K^+^-ATPase *β* subunit and *β*-actin) Kits (28990926 and 25006327, GE Healthcare) and detected in X-ray films (Kodak, MXG-Plus, Rochester, NY, USA) or in the G box transilluminator (Syngene, Frederick, MD, USA). Densitometry was performed for each protein (Image J, 1.37v Software, NIH Public Domain) and compared as the ratio of *β*-actin.

### 4.4. Morphology and Determination of Parietal Cell Index

For histological analyses, paraffin- embedded stomachs (fixation in 10% formaldehyde) were sectioned at four or six µm, using intervals of 30 µm to avoid double-quantification of parameters. After deparaffinization, sections were submitted to hematoxylin-eosin staining, and parietal cells (PC) were identified according to their morphology (roundness, central nucleus, acidophilic cytoplasm). Longitudinally sectioned areas were used for cell counting under light microscopy (Nikon, Tokyo, Japan) (X 800, using integrative eyepiece with grid-Kpl 12, Zeiss, Oberkochen, Germany) and to avoid the changes in gland depth and structure, fields close to the cornea and antral regions were not considered. The number of PC was determined in a total of 1000 epithelial cells and results were expressed as PC index (%). 

### 4.5. Histochemistry and Immunohistochemistry for Multiple Labeling

Following protocols described before [38], stomachs were processed to detect: (a) mucous neck cells(MNC) through histochemistry with *Griffonia simplicifolia* II lectin conjugated with fluorescein (FITC); (b) zymogenic cells (ZC) through immunohistochemistry against Mist1 and GIF; (c) transition cells (TC) as those labeled for GSII-FITC and Mist1 or GSII-FITC and Mist1 and GIF, as indicated.

Briefly, paraffin sections (fixation in 4 % formaldehyde) were obtained as above (4 µm non-serial sections) and placed on positively charged slides. For Mist1 and GIF immunostainings, after hydration with 0.3% Triton X-100 (10 min, RT) in phosphate buffered saline (PBS, 50 mmol/L, pH 7.2), sections were washed in PBS, and immersed in Tris-HCl (50 mmol/L, pH 9.0) for antigen retrieval (water bath in microwave for 5 min at 600W, and for 5 min at 300 W). Then, slides were washed in PBS, and goat anti-GIF (2 µg/mL) and monoclonal mouse anti-Mist1 (8 µg/mL) were added for incubation (4 °C, overnight) (sc 161643 and 80984, Santa Cruz Biotechnology). The development of reactions proceeded in the dark box, and after rinsing in PBS, Cy3-conjugated secondary antibody was used (30 µg/mL, 2 h, RT; 715-165-150, Jackson ImmunoResearch Laboratories) to detect Mist1. Next, donkey anti- goat conjugated with AlexaFluor 633 (20 µg/mL, 1 h, RT; A22082, Life Technologies, Carlsbad, CA, USA) was added to identify GIF labeling. Following these reactions, sections were incubated with GSII-FITC (20 µL/mL, 2 h, RT; Vector, Burlingame, CA, USA). Finally, slides were carefully washed in PBS and nuclei were counterstained with 4’,6-diamidino-2-phenylindoledehydrochloryde (DAPI, 0.5 µg/mL, 2 min; Life Technologies). Slides were mounted in Mowiol (Calbiochem-Merck, La Jolla, CA, USA). For negative controls, primary antibodies were omitted, separately, and sections were compared. 

Sections obtained from the gastric mucosa of S and EW rats at 18 and 60 days were studied under fluorescence microscopy (Axioscope 2, Zeiss) and images from central areas in corpus region, where glands were sectioned longitudinally were acquired at X 400 (Zen 2011 Software, blue edition, Zeiss). From these images, we used five fields/ rat to estimate the number of cells/field from each population: MNC, TC and ZC, considering that for TC, two stages of transition (+GSII + Mist1 and +GSII + Mist1 + GIF) were used. Photomicrographs in high resolution were acquired using Zeiss LSM 780-NLO confocal microscope (CEFAP ICB USP) with argon laser and 2 excitation lasers HeNe (458, 488, 514, 543, and 633 nm).

### 4.6. Statistical Analyses

No statistical test was used to predict sample size. Animals were randomized into S and EW groups at 15 days. Biological (*n* of rats) and technical replicates are indicated. Results are shown individually according to the parameter and the means ± SD are indicated. One-way ANOVA was used to detect variation in gene expression during gastric growth. In order to identify the effects of EW on gene expression, we built a heat map. Additionally, to investigate the relationships among the variables and to visualize meaningful patterns in the gene expression data, we performed a principal component analysis (PCA). Differences between S and EW groups were compared in each age by unpaired Student *t* test and were considered significant at *p* < 0.05. ANOVA, Student *t* tests and heatmap were performed using GraphPad Prism 8.0 software (GraphPad Software Inc., La Jolla, CA, USA). Thumb’s test for normal distribution and PCA were conducted using RStudio software 1.0.153 (RStudio: Integrated Development Environment for R, Inc., Boston, MA, USA). 

### 4.7. Ethics Statement

All procedures were performed according to the guidelines of National Council of Ethics with Animals (CONCEA) that are in accordance with the Animal Research: Reporting of In Vivo Experiments (ARRIVE) guidelines (www.nc3rs.org.uk/ARRIVE), and the protocols were approved by the Ethical Committee for Animal Use from Institute of Biomedical Sciences at University of Sao Paulo (CEUA ICB USP certificates18/2015 and 115/2017 that were respectively approved in 02/23/2015 and 10/31/2017). Moreover, experiments were conducted in order to minimize the number of animals and to allow best conditions during treatment.

## Figures and Tables

**Table 1:** Primers used for detection of gene expression after qPCR in ontogenic analyses.

Encoded Protein	Gene	Forward	Reverse
**GAPDH**	*Gapdh*	AGTGCCAGCCTCGTCTCATAG	TAACCAGGCGTCCGATACG
**H^+^/K^+^ ATPase**	*Atp4a*	TGCCCATCCGGTTCCA	TCCGGATCTCATCATAGACAAA
**Mucin 6**	*Muc6*	TACCTCTCACAGGAAGGACTACCAT	TCGTGTACTTGTTTTAGGTGGTGCTA
**Mist1**	Bhlha15 *	GAACTTGTGCTTGGTCCATCCT	TCCCTATCCTGCGTTCACAAC
**GIF**	*Gif*	TGTGGCCCTGATCATGAAGT	TTGTTTATGGTGTATATGACTGTGA
**Pepsinogen C**	*PgC*	CTGGCTTCTTTGGCTATGACACT	CTCATTCTCACTCAGGCCAAACT

* Primers also used for comparison between S and EW groups.

**Table 2:** Taqman^®^ probe assays used for detection of gene expression after qPCR.

Encoded Protein	Gene	Assay ID
**Beta-actin**	*Actb*	Rn 00667869_m1
**ATPase H^+^/K^+^**	*Atp4b*	Rn 00560844_m1
**Mucin 6**	*Muc6*	Rn 01759814_m1
**GIF**	*Gif*	Rn 00567702_m1
**Pepsinogen 5a**	*Pg5a*	Rn 00572739_m1
**Pepsinogen C**	*Pgc*	Rn 00590984_m1

## Abbreviations

S	Suckling
EW	Early weaning
